# RFID Tracking of Sublethal Effects of Two Neonicotinoid Insecticides on the Foraging Behavior of *Apis mellifera*

**DOI:** 10.1371/journal.pone.0030023

**Published:** 2012-01-11

**Authors:** Christof W. Schneider, Jürgen Tautz, Bernd Grünewald, Stefan Fuchs

**Affiliations:** 1 Institut für Bienenkunde, Department of Biological Science, Goethe-University, Frankfurt am Main, Germany; 2 BEEgroup, Biocentre, University of Würzburg, Würzburg, Germany; Université Paris 13, France

## Abstract

The development of insecticides requires valid risk assessment procedures to avoid causing harm to beneficial insects and especially to pollinators such as the honeybee *Apis mellifera*. In addition to testing according to current guidelines designed to detect bee mortality, tests are needed to determine possible sublethal effects interfering with the animal's vitality and behavioral performance. Several methods have been used to detect sublethal effects of different insecticides under laboratory conditions using olfactory conditioning. Furthermore, studies have been conducted on the influence insecticides have on foraging activity and homing ability which require time-consuming visual observation. We tested an experimental design using the radiofrequency identification (RFID) method to monitor the influence of sublethal doses of insecticides on individual honeybee foragers on an automated basis. With electronic readers positioned at the hive entrance and at an artificial food source, we obtained quantifiable data on honeybee foraging behavior. This enabled us to efficiently retrieve detailed information on flight parameters. We compared several groups of bees, fed simultaneously with different dosages of a tested substance. With this experimental approach we monitored the acute effects of sublethal doses of the neonicotinoids imidacloprid (0.15–6 ng/bee) and clothianidin (0.05–2 ng/bee) under field-like circumstances. At field-relevant doses for nectar and pollen no adverse effects were observed for either substance. Both substances led to a significant reduction of foraging activity and to longer foraging flights at doses of ≥0.5 ng/bee (clothianidin) and ≥1.5 ng/bee (imidacloprid) during the first three hours after treatment. This study demonstrates that the RFID-method is an effective way to record short-term alterations in foraging activity after insecticides have been administered once, orally, to individual bees. We contribute further information on the understanding of how honeybees are affected by sublethal doses of insecticides.

## Introduction

**Figure 1:**
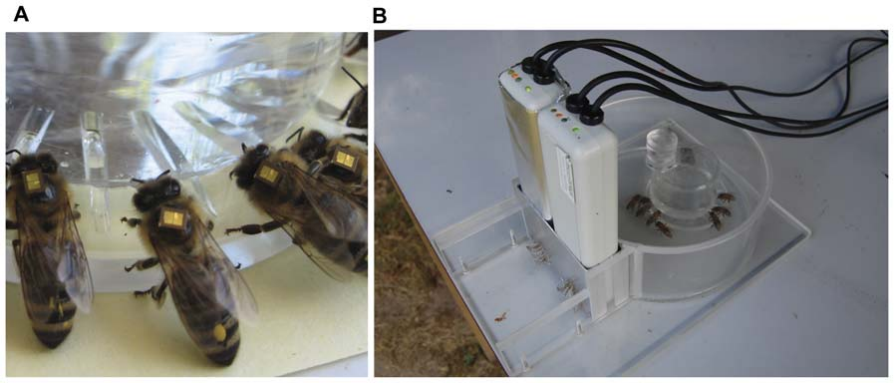
Automatic registration at the feeder. (**A**) The RFID-transponder attached to the thorax shield of honeybees allows tracking of the foraging activity with RFID-readers positioned at hive entrance and at the feeder. (**B**) Bees foraging from an artificial feeder placed in a feeder compartment. The bar-shaped scanners at the entrance of the feeder compartment detect every passage of a labeled honeybee, when it passes through specially crafted bee tunnels (see also Figure S4).

Sublethal effects have been described as effects on physiology and behavior of an individual that has been exposed to a pesticide without directly causing death [1]. For honeybees, exposure to sublethal insecticide doses can have an influence on their learning ability, orientation, foraging, or brood care [2]. In their role as pollinators honeybees interact with plants that are targeted by insecticide application. Therefore, standard guidelines have been developed to assess the risk of these substances [3]–[6]. These tests include toxicity evaluations on adult bees by cage-, tunnel-, and field experiments, mainly observing mortality. Residual toxicity is considered to be less important, due to the fact that these guidelines deem the main exposure way to be by spraying application. Taking into consideration the systemic properties of insecticides like neonicotinoids, seed dressing has become a major practice for plant protection. It effectively reduces the amount of insecticides used on agricultural crop land by up to 99% (Bundesamt für Verbraucherschutz und Lebensmittelsicherheit, BVL, Germany) and is supposed to reduce health risks by minimizing interaction of the active ingredients with the surrounding environment. Nevertheless, the honeybee can be exposed to these substances by two main exposure routes: contact and oral exposure. When considering oral ingestion, honeybees can be exposed in different ways including nectar, pollen, and guttation water. Guttation water, an excretion of xylem water at the leaf margins, was recently discovered to hold high residues of neonicotinoid substances (imidacloprid, clothianidin, thiamethoxam) when collected from treated corn seedlings (*Zea mays* L.) [7]. It still remains unclear though, if water foragers collect guttation drops from seed dressed plants and, if they do, how these drops affect the bees. The residues of imidacloprid and clothianidin found in pollen and nectar of seed dressed sunflowers (*Helianthus annuus* L.) clearly range at a sublethal level [8], [9]. Field relevant doses of imidacloprid in sunflowers and oilseed rape were estimated to be around 0.13 ng and 0.023–0.03 ng, respectively [10]. In the laboratory, impairments of insecticides on honeybee learning are commonly investigated using proboscis extension reflex (PER) conditioning [11]–[15]. This paradigm simulates the conditioning process of memorizing a floral cue, e.g. odor, and associating it with the reward nectar and pollen [16]. Outside of the lab the influence of sublethal insecticide doses is investigated by analyzing the foraging behavior at an artificial feeder containing sugar water as a nectar substitute and/or by analyzing the homing ability of the treated bees [17]–[20], but the observations are time-consuming and the information provided is limited. Therefore, we wanted to apply a feeder task combined with RFID labeling (**Fig. 1A**). RFID-labeling was introduced in honeybee research by Streit et al. in 2003 [21] and was also used for investigations of other hymenopterans [22], [23] to obtain detailed information on foraging behavior with little effort and at reasonable cost. Independently from this study, Decourtye *et al.* [24] developed a similar feeder task which, like the approach in this study, was based on two sets of separate direction-sensitive reading devices positioned in front of the hive entrance and in front of a compartment containing an artificial feeder. They showed that the phenylpyrazole insecticide fipronil reduced the number of foraging flights and prolonged the duration of homing flights for up to three days.

While Decourtye *et al.* investigated for effects of fipronil on longevity, as well as the number and duration of the homing flight, our study focused on the effects of the neonicotinoid insecticides imidacloprid and clothianidin. The latter has been identified as the main cause for a massive bee intoxication incident in Germany in the spring of 2008, resulting from poor seed dressing quality. With two reading devices at hive and feeder (**Fig. 1B**), respectively, we observed the number of foraging trips from the hive to the feeder, the duration of these foraging trips, and the time interval a bee spent inside the hive between foraging trips. In addition, we obtained detailed information on foraging trip phases by dividing the trip duration into three sections: flight time to the feeder, duration of stay at the feeder, and flight time back to the hive. Sublethal effects of imidacloprid on honeybee foraging behavior have been investigated before by other methods [18]–[20]. Therefore, the number of tests with the imidacloprid have been limited to two trials in the summers of 2009 and 2010, as well as an additional pre-test in 2008 (data of pre-test shown in Figure S1) in order to calibrate and validate the experimental design. Clothianidin-related effects on foraging are not yet reported to our knowledge. Thus, we focused this study mainly on this particular substance. After the bees had been released at the feeder site following one-time insecticide administration, we analyzed the foraging behavior during three-hour periods immediately after and between 24 h and 48 h after treatment. A maximum of five different treatment groups, including a control group, were tested simultaneously. Thus, we provide further information on sublethal influences of insecticides after oral administration to individual honeybee foragers and corroborate the suitability of the RFID method for this purpose.

## Results

This study included 10 independent trials, two of which were performed with imidacloprid and eight of which were performed with clothianidin. The limited number of imidacloprid trials is due to the fact that imidacloprid is well known to cause sublethal effects on foraging behavior. Thus, these trials mainly had a calibration and validation purpose for our experimental design. The distance between the hive and the feeder in each trial was 7 meters. RFID-tagged bees (*Apis mellifera carnica*) undertook 29610 foraging flights to the feeder. The median total duration an untreated bee needed for a foraging trip in the different tests, from leaving the hive until reentering it, lasted between 107 and 130 seconds. The median flight time to the feeder lasted between 7 and 11 seconds, the median flight time back to the hive took between 9 and 12 seconds, and the median period of time a bee spent at the feeder was between 76 and 110 seconds. Between two foraging flights the bees spent a median time of 95–111 seconds inside of the hive. The variation in the durations is explained by the different weather conditions during the conduction of the tests.

### 1. Proportion of bees returning to the hive after treatment

In the trials conducted with imidacloprid, all or almost all bees of the control groups and the groups treated with doses up to 3 ng returned to the hive after post-treatment release at the feeder, but only a quarter of the bees returned after administration of 6 ng (controls, 0.15 and 1.5 ng: 100%; 3 ng: 95%, 6 ng 25%). Among the bees treated with 3 ng and 6 ng imidacloprid that were not directly flying to the hive, we observed reduced mobility, followed by a phase of motionlessness with occasional trembling and cleaning movements (Movies S1 and S2).

In the trials conducted with clothianidin, all of the control- and 0.05 ng-bees, and 94.4% of the 0.5 ng-bees returned to the hive during a three-hour observation period immediately after treatment. From the bees treated with 1 ng, only 73.8% returned to the hive, and only 20.6% returned after the uptake of 2 ng. We repeatedly observed abnormal reactions after the release at the feeder site following the administration of 1 ng and 2 ng clothianidin. Bees were moving around with an awkwardly arched abdomen, sometimes followed by a phase of turning upside down and lying on the back with paddling leg movements (Movie S3 and S4). Regardless of the administered substance, bees that did not return to the hive within the three-hour period immediately after treatment were neither registered again at the hive nor at the feeder during the following days.

### 2. Feeder visits

**Figure 2:**
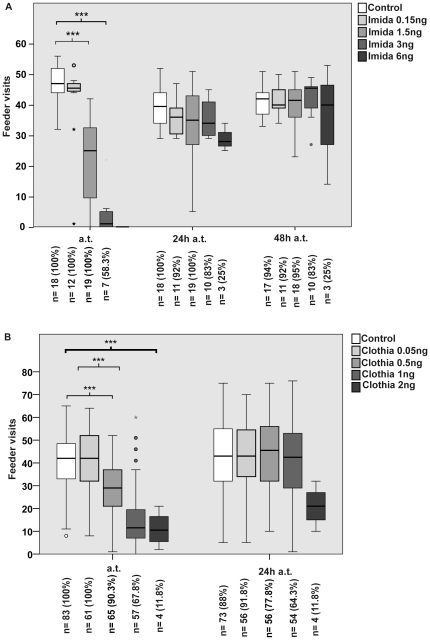
Changes in the frequency of feeder visits after treatment with imidacloprid or clothianidin. During 3-hour observation periods, we recorded the frequency of visits at the feeder immediately after treatment (a.t.) and up to 48 h after treatment (24 h a.t., 48 h a.t.). Values inside the bars: n = Number of bees returning to the feeder after treatment, % = (bees returned/bees treated)*100, (**A**) Oral administration with 0.15 ng imidacloprid did not affect the visit frequency per bee significantly, while 1.5 and 3 ng imidacloprid led to a significant reduction of feeder visits compared to the controls during the first three hours immediately after treatment. Not all bees treated with 3 ng reappeared at the feeder immediately after treatment, but almost all returned regularly after 24 h. No bees treated with 6 ng imdacloprid returned in the first three hours of observation after treatment. After 24 h only three of twelve bees returned to forage from the feeder. (**B**) The frequency of visits was not affected negatively after administration of 0.05 ng clothianidin, while treatment with 0.5 ng, 1 ng, and 2 ng clothianidin reduced the frequency of visits significantly compared to the control group during the first three hours immediately after treatment. As shown here, only 67.8% and 11.8% of the bees treated with 1 ng and 2 ng, respectively returned to forage at the feeder. The missing bees were not registered again during the experiments. The significant reductions to the visit frequency by both substances did not persist on the following day. * = p<0.05, ** = p≤0.01, *** = p≤0.001.

The number of feeder visits per bee that was detected for the variously dosed imidacloprid groups during the different observation periods over 48 h is shown in **Figure 2A**. The median number of feeder visits for bees treated with 1.5 ng and 3 ng imidacloprid compared to the control bees (n = 18) during the three-hour observation period after administration was reduced by 47% and 98%, respectively (n = 19, p_1.5 ng_<0.001; n = 10, p_3 ng_<0.001, Mann-Whitney-U-test). Not all bees treated with 3 ng reappeared at the feeder immediately after treatment, all but two returned regularly after 24 h. Bees treated with 6 ng, which had re-entered the hive after release, did not visit the feeder until 24 h after treatment.

Administration of 0.5 ng and 1 ng clothianidin resulted in a significant reduction of the number of feeder visits per bee compared to the control group (n = 83). During the three-hour observation period after treatment the visit frequency was reduced by 31% and 71%, respectively (n = 65, p_0.5 ng_<0.001; n = 57, p_1 ng_<0.001, Mann-Whitney-U-test, **Fig. 2B**). For the bees that returned to the feeder after administration with 2 ng clothianidin the number of visits was reduced by 74% (n = 4, p_2 ng_<0.001). The lowest doses used for both substances, 0.15 ng for imidacloprid and 0.05 ng for clothianidin, had no effect on the number of feeder visits.

Twenty-four hours after administration, no verifiable effect on the average number of feeder visits was detected for any of the treatment groups except for the 6 ng imidacloprid (p_6 ng_ = and the 2 ng clothianidin group (p_2 ng_ = 0.013).

### 3. Duration of and time interval between foraging trips

**Figure 3:**
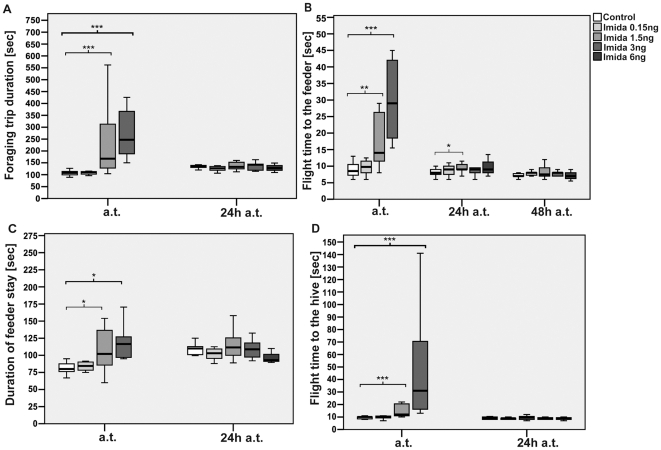
Influence on foraging trip duration and its different phases after treatment with imidacloprid. Plotted were the median times in seconds for every bee during 3-hours observation periods immediately after treatment (a.t.) and 24 h after treatment (24 h a.t.). * = p<0.05, ** = p≤0.01, *** = p≤0.001. (**A**) Imidacloprid: Bees treated with 1.5 ng and 3 ng imidacloprid spent more time outside of the hive for a foraging trip compared to the control group during the first three hours immediately after treatment. These effects were not persistent after 24 h. (**B**) We observed significantly prolonged flights to the feeder and (**D**) back to the hive during the three hour observation period after oral administration, for bees treated with 1.5 and 3 ng imidacloprid compared to the control group during the first three hours immediately after treatment. (**C**) Bees treated with 1.5 and 3 ng imidacloprid spent more time inside the feeder compartment compared to the control group during the first three hours after treatment. This effect was not found to be persistent 24 hours after administration.

Administration of 1.5 ng and 3 ng imidacloprid substantially prolonged the median duration needed for a single foraging trip by 50% and 130% (p_1.5 ng_≤0.001; p_3 ng_<0.001, Kruskal-Wallis-Test followed by Mann-Whitney-U-Test), respectively during the first three-hour observation period (**Fig. 3A**). In the experimental groups treated with 1.5 ng or 3 ng imidacloprid, the median flight time to the feeder was prolonged by 64.7% and 241.1%, respectively (p_1.5 ng_<0.01; p_3 ng_<0.001, see also **Fig. 3B**). The time spent at the feeder was prolonged by 27.5% and 45.6%, respectively (p_1.5 ng_<0.05; p_3 ng_<0.05, see also **Fig. 3C**), and the median flight time from the feeder to the hive was prolonged by 20% and 210%, respectively (p_1.5 ng_<0.01; p_3 ng_<0.001, see also **Fig. 3D**). In addition, the median duration the bees spent within the hive between foraging flights was significantly prolonged by 33% and 993% after administration of 1.5 ng (p_1.5 ng_<0.001) and 3 ng (p_3 ng_<0.001), respectively, as compared to the control bees. This effect was particularly pronounced during the first and second time interval inside the hive between foraging trips after administration of 3 ng (1^st^: 972.2%, p_3 ng_<0.001; 2^nd^: 1077.7%, p_3 ng_<0.05) in relation to the controls. Administration of 1.5 ng did not affect the first, but prolonged the second in-hive stay (33%, p<0.01). Twenty-four hours after administration of 1.5 ng we still found prolonged flight times to the feeder but the effect was not as pronounced as the day before. None of the other described effects persisted during the following days.

**Figure 4:**
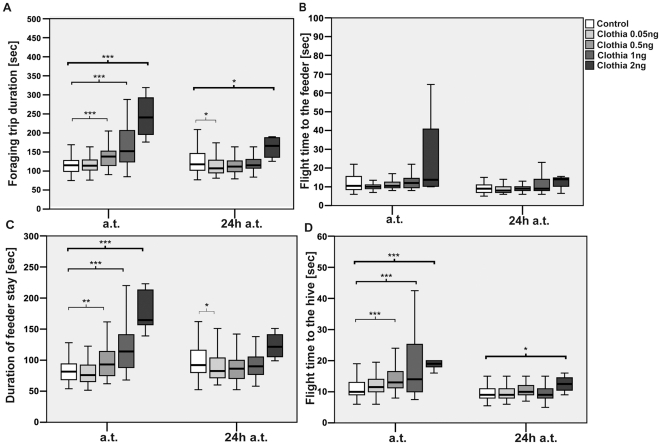
Influence on foraging trip duration and its different phase after treatment with clothianidin. Plotted were the median times in seconds for every bee during 3-hours observation periods immediately after treatment (a.t.) and 24 h after treatment (24 h a.t.). * = p<0.05, ** = p≤0.01, *** = p≤0.001. (**A**) After bees were treated with 0.5 ng, 1 ng, and 2 ng clothianidin their median time spent outside of the hive was significantly prolonged compared to the control group. On the following day we observed slightly but significantly shorter foraging trips by the bees treated with 0.05 ng compared to the control. Foraging trips by the bees treated with 2 ng clothianidin were still found to be significantly prolonged after 24 hours, though not as profound compared to the day before. (**B**) Treatment with clothianidin, regardless of the dose, showed no significant effect on flight time to the feeder. (**C**) Immediately after treatment with 0.5 ng, 1 ng and 2 ng clothianidin, bees spent more time inside the feeder compartment compared to the control group. Twenty-four hours after treatment we observed significantly shorter feeder visits for bees treated with 0.05 ng when compared to the control, while no significant difference was observed for bees treated with the other doses. (**D**) Bees treated with 0.5 ng, 1 ng, and 2 ng needed significantly longer to fly back to the hive compared to the controls during the three hour observation period immediately after treatment. After 24 h bees treated with 2 ng still needed significantly longer than the control group when returning to the hive, though the difference was not as profound compared to the day before.

**Figure 5:**
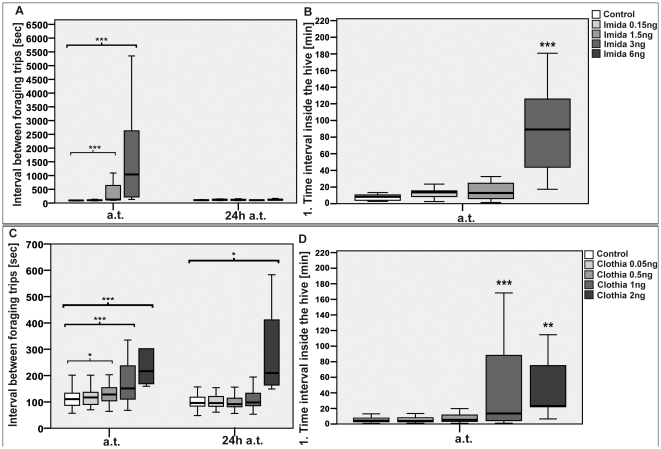
Time interval between foraging trips after treatment with both insecticides. Plotted was the median period spend inside the hive between two foraging trips and the duration of the first in-hive stay for every bee (in minutes) immediately after respective administration of one of the two insecticides.* = p<0.05, ** = p≤0.01, *** = p≤0.001. (**A**) In the three hour observation period after treatment with 1.5 ng and 3 ng imidacloprid and (**C**) 0.5 ng, 1 ng and 2 ng clothianidin, these bees needed significantly longer to fly out again after returning from for the subsequent foraging trip compared to the control groups. (**B**) Administration of 3 ng imidacloprid led to a significantly prolonged first stay inside of the hive. (**D**) Bees that were treated with 1 ng and 2 ng clothianidin had longer first in-hive stays compared to the controls.

The median duration of a foraging trip of bees treated with 0.5 ng, 1 ng, and 2 ng clothianidin was significantly prolonged by 20% (p_0.5 ng_<0.001), 32.2% (p_1 ng_<0.001), and 109.3% (p_2 ng_<0.001) compared to the control bees during the three-hour observation period immediately after administration (**Fig. 4A**). In contrast to the administration of imidacloprid, treatment with clothianidin, regardless of the dose, had no verifiable effect on the median flight time to the feeder (**Fig. 4B**). Immediately after administration of 0.5 ng, 1 ng, and 2 ng, the time spent at the feeder was prolonged by 14.1% (p_0.5 ng_<0.01), 39.9% (p_1 ng_<0.001), and 101.8% (p_2 ng_<0.001) (see also **Fig. 4C**). The median flight time back to the hive was prolonged by 30% (p_0.5 ng_<0.001), 40% (p_1 ng_<0.001), and 90% (p_2 ng_<0.001), respectively (**Fig. 4D**). The median time interval of an in-hive stay between foraging trips was prolonged by 15.8% for 0.5 ng (p_0.5 ng_<0.05), by 36.7% for 1 ng (p<0.001), and by 95.9% for 2 ng (p_2 ng_<0.001) (**Fig. 5C**). The first and second stay inside the hive immediately after treatment were substantially prolonged after administration of 1 ng (1^st^: +192.4%, p_1 ng_<0.001; 2^nd^: +82.2%, p_1 ng_<0.001) and 2 ng (1^st^: 400%, p_2 ng_<0.01 and 2^nd^: 190.7%, p_2 ng_<0.05) compared to the control bees (**Fig. 5D**). Administration of 0.5 ng did not affect the first, but prolonged the second in-hive stay by 71.9% (p_0.5 ng_<0.05). No detrimental effects on the parameters above were found for 0.05 ng. However, 24 h hours after oral administration of 0.05 ng, bees needed less time for a foraging trip (−9.1%; p_0.05 ng_<0.05) by spending shorter periods of time at the feeder (−8.9%; p_0.05 ng_<0.05). Twenty-four hours after treatment with 2 ng, we still observed a prolonged median duration for a foraging trip (+41.3, p_2 ng_<0.05), for a flight back to the hive (+38.9%, p_2 ng_<0.05), and for the time interval spent inside of the hive between foraging trips (+118.2%, p_2 ng_<0.05) No significant effects were detectable after 24 h for the other doses used.

## Discussion

Our study used the RFID-technology to analyze the impact of insecticide compounds on honeybee foraging behavior. Decourtye *et al.* already showed that fipronil at doses of 0.3 ng/bee reduced the number of foraging flights to the feeder and prolonged the duration of the homing flight [24]. These effects were observable for up to three days. Similar effects were found for the compounds used in this study. In contrast to Decourtye *et al.*, who conducted their tests under semi-field conditions, the described trails were conducted under field conditions, allowing the test colonies to normally provide themselves with necessary additional resources including pollen, water, and propolis. Furthermore, since the test is designed to detect effects on individual bees, the tested bees were fed defined amounts of the pesticide individually with the differently spiked sugar solutions instead of bulk feeding them in a cage.

By obtaining data describing a minimum of six different parameters of foraging behavior, sub-lethal effects for both substances, imidacloprid and clothianidin, used in this study were detected. Impairments were detected at doses of 1.5 ng imidacloprid per bee, which would equal a concentration of around 115 ppb (parts per billion) in nectar. These results are in agreement with previous studies, which tested the effect of imidacloprid on homing and foraging behavior [18]. Yang *et al.* [20] found effects on foraging behavior at concentrations as low as 50 µg/L (40–50 ppb). These documented concentrations are still more than twenty-five to fifty times higher than the residues found in the nectar of sunflowers (*Helianthus*, 1.9 ppb) [8]. Treatment with the lowest dose of imidacloprid (0.15 ng; 11.5 ppb), which is about five-fold higher than any residues found in nectar, had no recognizable effect on foraging behavior. Nevertheless, bees may be exposed to almost 100-fold higher doses than tested in our trials, as shown in a study concerning the insecticide contamination of guttation drops, xylem fluids that are excreted at the leaf margins [7] in seed dressed crops. It remains unclear, though, if water foragers collect these fluids in the field.

This is the first study on foraging behavior of honeybees that presents sub-lethal effects after acute oral treatment with clothianidin. Dosages of 0.5 ng (38 ppb) negatively influence the foraging behavior and low dosages (0.05 ng; 3.8 ppb) can have effects on certain aspects of foraging behavior even if they did not have any significant effects on the number of feeder visits or on the total foraging time. Clothianidin elicited detrimental sub-lethal effects at somewhat lower doses (0.5 ng/bee) than imidacloprid (1.5 ng/bee). Bees disappeared at the level of 1 ng for clothianidin, while we could register the first bee losses for imidacloprid at doses exceeding 3 ng. This indicates a stronger impact of clothianidin compared to imidacloprid, which is in agreement with previous reports that both oral [7] and contact toxicity [25] levels are lower for clothianidin.

Both neonicotinoid insecticides are known to be partial agonists for different types of the insect nicotinic acetylcholine receptor (nAChR) [26]–[30]. In vitro experiments that observed the action of imidacloprid and clothianidin on native nAChRs of cholinergic neurons from *Drosophila* larvae [31] and nAChRs of the terminal abdominal ganglion neurons of the American cockroach [32] showed greater agonist efficacy of clothianidin compared to imidacloprid. A similar effect on cholinergic neurons in adult honeybees could be an explanation for our findings that clothianidin elicits detrimental effects at lower doses than imidacloprid.

In general, both substances led to similar effects on the observed foraging parameters. The only difference was found when investigating the flight time to the feeder. Bees treated with imidacloprid needed more time to fly to the feeder while no effect on this phase was observed after administration of clothianidin. Relating this to the symptoms observed after administration of higher doses of both substances it could be another indication for differences in their efficacy. Higher doses of imidacloprid (≥3 ng) led to reduced movement, eventually leading to immobility and trembling movements, which is in accordance to previously reported doses of ≥2.5 ng by Lambin *et al.* [33]. This might have occurred to some degree in the lower doses as well, but escaped visual observation. Higher doses of clothianidin (≥1 ng), in contrast resulted in an arched abdomen, which did not reduce the mobility of the bees like imidacloprid did. Girolami *et al.* [7] reported that when their abdomens were arched, the bees still retained their flying capability. Taking this into consideration, it could explain why the detrimental effect on flight behavior by clothianidin was less pronounced compared to imidacloprid, though still significant compared to the controls. Furthermore, it might be hypothesized that imidacloprid and clothianidin aim at differing targets, i.e. different subtypes of receptors located on pre-motoneurons and motoneurons of honeybees, though their cell physiological actions are still unknown. This was already shown in a study of Thany [34] for DUM-(dorsal unpaired median) neurons isolated from the cockroach *Periplaneta americana*. Here clothianidin was found to act on imidacloprid-sensitive and -insensitive nAChR subtypes. DUM-neurons are known for their neuromodulatory role in altering the performance of motor patterns and are thought to be homologous to VUM (ventral unpaired median)-neurons [35], [36] of honeybees because of their similar morphology and physiology.

An increase in motor activity observed in a study by Lambin *et al.* after topical application of 1.25 ng imidacloprid and subsequent introduction into an open-field-like apparatus [33]. The orally administered dosages of imidacloprid in our study did not seem to increase motor activity at the feeder site.

Both substances led to a longer 1^st^ and 2^nd^ period of stay inside the hive before returning to the feeder. This is likely due to a prevailing toxic effect on the bees while they were inside the hive. They remained in the hive until the effect ceased and they were able to fly out again. This is consistent with the fact that bees that did not return to the hive after treatment were not registered again, but the majority of bees that made it back to the hive returned to the feeder later on.

In conclusion, we think that the method of RFID aided feeder tests could be of considerable help concerning risk assessment of insecticides. Subsequent to initial mortality tests to determine the LD_50_ of an insecticide, the RFID-method could be used in field-like tests to investigate possible sublethal effects of doses thought to be non-hazardous for honeybees. Furthermore, we think that the sensitivity would be increased considerably by higher distances between hives and feeder, which will be explored in further experiments.

## Materials and Methods

To investigate foraging behavior, a classical behavioral feeding experiment was combined with modern monitoring technology. Our behavioral paradigm was to train the bees to forage at an artificial food source and monitor their performance. All tests were conducted during the summers of 2009 and 2010 at a research compound situated about 370 meters east of the Institut fuer Bienenkunde in Oberursel, Germany. Each trial included several training steps, individual pesticide treatment and a subsequent observation period of up to 48 hours. One week was needed to conduct a single test.

### 1. Monitoring by RFID technology

For exact and detailed monitoring of individual bee's foraging behavior, bees were labeled with RFID (radio frequency identification) transponders (mic3-TAG 64bit read only, carrier frequency: 13.56 MHz, microsensys GmbH, Erfurt, Germany), each holding a unique ID number. The RFID-Transponder was attached to the bees on the dorsal part of their thorax one day before insecticide application. The dimensions of the transponders were 2×1.6×0.5 mm, and the weight was approximately 4 mg. For labeling the bees we used commercially available devices for marking queens (Carl-Fritz-Imkereifachhandel, Mellrichstadt, Germany) consisting of plastic tubes closed by an elastic mesh closes at one end of the tube. Bees were gently pushed against the net with a soft foam plastic covered plunger, such that the tags could be glued to the upper part of the thorax with a drop of shellac through a mesh hole, where it was allowed to dry for 15 to 20 min. All tags used in one trial were checked as to their functioning with a handheld USB-Penreader (iID® PEN mini USB, microsensys, Erfurt, Germany) and their IDs were saved before attaching them to the bees.

For tracking bee movements, specifically designed scanners (Model: 2k6 HEAD, memory: 512 kByte, Controller-Update v27, Hardware-Update v 2.4, microsensys GmbH, Erfurt, Germany) were positioned in front of the hive entrance.. As the reading distance of this RFID system is limited to approximately 4 mm and the tag, fixed on the bee, has to be aligned facing upward, bees were to enter the colonies through tunnels whose specific shape of cross-section was to ensure that bees would not pass upside-down or sideways (see also **Fig. S2**). For each entry, two parallel tunnels, each equipped with a separate scanner were used. For the automatic registration of labeled bees at the feeder site, we developed a custom-made feeder compartment from acrylic Plexiglas® (dimensions: 229×165×55 mm). In order to forage from the feeder, each bee had to enter the compartment through an identical tunnel-system, as used at the hive entrances.

With these reading devices it was possible to track individual bees at the hive entrance or the feeder site, respectively, to receive an exact time-stamp (date and time) when the bees were registered, and to determine the direction the bees were heading, either departing or arriving. From the readers at the colony entrance alone, total durations of foraging flights and the duration of stays inside the colony between flights could be determined. Using additional readers at the feeder sites allowed to determine the numbers of visits to the feeder, but also to differentiate the durations of the different phases of a foraging trip which were (i) the time interval between leaving the hive and entering the feeder compartment termed “flight time to the feeder”, (ii) the time interval between entering and leaving the feeder compartment (“time spent at the feeder”) and (iii) the time interval between leaving the feeder and entering the colony (“flight time to the hive”).

### 2. Foraging behavior setup

#### Colonies

In each year, bees of an *Apis mellifera carnica* breeder line were housed in a nucleus bee hive (Mini-Plus, Bienenzuchtbedarf Heinrich Holtermann GmbH & Co KG, Brockel, Germany) containing 6 mini combs (approx. 248×159 mm) and about 2000 bees. The hive entrances were marked by black geometrical shapes (triangle, circle, or square) on a white background to provide visual guidance for the departing and returning bees.

#### Feeders and training

A feeder, similar to the design of Renner (1959) [37], filled with an odorless 2 M sucrose solution was placed on a table 7 m away from the hive entrance. To offer the bees additional visual and olfactory cues to be associated with the food source, a square-shaped 75×75 mm yellow wax patch from comb foundations was placed beneath the feeder. Departing worker bees of unknown age were caught at the hive entrance in 20 ml snap cap bottles, carried to the feeder where they were released so that they could collect sugar solution if it seemed attractive enough to them. Bees returning to the feeder after flying back to the colony were color-marked on the abdomen. On a given day, between 90 and 100 individuals were color-marked. After marking, unmarked newcomers to the feeder were caught and disposed of to avoid crowding.

Unlike the registration tunnels in front of the beehive, which the bees learned to pass of their own accord when leaving and entering the colonies, a special training was required to guide our color-marked foragers to the feeder within the feeder compartment (**Fig. 1B**). After the bees had learned to forage from the freely accessible feeder, the feeder together with the wax patch was placed inside the feeder compartment for about 30 minutes. Then the compartment was covered by the lid so that bees entered through the entry opening still not equipped with the registration tunnels, which were inserted 30 min later as the sole openings. To help first foragers until traffic was established, these were additionally guided into the tunnels by wax markings and sugar trails, and the compartment was covered to exclude light except from the tunnels to help them to learn to leave the compartment.

### 3. Administration of insecticides

We were interested to see if the sublethal influence of certain insecticides altered the foraging parameters described above. For our tests, we used imidacloprid (powder form, Bayer AG, Leverkusen, Germany) and clothianidin (powder form, Sigma-Aldrich, St. Louis, Missouri, USA). Both substances were applied orally, dissolved in 2 M sucrose solution. The solubility of both neonicotinoids in water (imidacloprid: 0.51 g/L; clothianidin: 0.327 g/L) made it necessary to pre-dissolve 10 mg of both substances with 1 ml of acetone before mixing them with distilled water and thereby gaining a stock solution of 1 mM. For both substances, dilution series were done to obtain concentrations in 2 M sucrose solution for imidacloprid of 0.06, 0.6, 1.2 and 2.4 µM which are equivalent to dosages per 10 µL of 0.15, 1.5, 3 and 6 ng. The lowest dose is in accordance with the estimated dose found in the nectar of seed treated sunflowers [10]. For clothianidin, those concentrations were 0.02, 0.2, 0.4 and 0.8 µM. These are equivalent to dosages per 10 µL of 0.05, 0.5, 1 and 2 ng. Controls were fed with 2 M sucrose solution containing an equivalent of acetone. The percentage of acetone did not exceed 0.01% (v/v).

In every trial, the previously labeled bees were caught at the feeder site immediately after landing with the same type of marking tube already used in the RFID-labeling process and were fed individually with different dosages of the tested insecticides. The sugar solution was offered in a cap from a 1.5 ml Rotilabo® micro centrifuge tube (Carl Roth GmbH, Karlsruhe, Germany), which was embedded in the foam plastic of the plunger, thereby serving as a small feeding trough. In order to assign the bees to the different dosage groups, the bees were caught and allocated to the experimental groups as follows: The first bee caught was put into group 1 (e.g. control), the second bee into group 2 (dosage x), the third bee into group 3 (dosage y), and so on. This pattern was repeated until all labeled bees were caught and assigned to one of the experimental groups. The maximum number of bees per dosage group was 12. The bees were kept isolated in the tubes for 20 min to avoid trophallaxis with other bees and to observe the possibility of regurgitation. After the treatment, the bees were released at the feeder site.

### 4. Data analysis

The reader data were read out with software supplied by microsensys GmbH, Erfurt, Germany, and were imported into statistical software (SPSS Statistics 17.0, SPSS Inc., Chicago, Illinois, USA). We used self-written algorithms to (i) filter and erase rapid succession registrations at the same reader antenna, which occurred when a labeled bee lingered beneath it for too long, (ii) calculate the time of a foraging trip and its different phases, and (iii) analyze the number of feeder visits per bee during the three-hour observation period. Since the number of feeder visits per bee and the times for the different foraging phases were not found to be normally distributed, non-parametrical Kruskal-Wallis- and Mann-Whitney-U-tests were used to the different treatment groups to the control. The null hypothesis was rejected at the 5% -level (p<0.05).

## Supporting Information

Figure S1 **Results of pre-test conducted with a handheld USB-Pen to detect bees at the feeder site.** (**A**) The number of visits at the feeder site was significantly reduced, (B) the median total duration for a single foraging trip and the first time interval spent inside the hive were significantly prolonged compared to the control (n = 10) for bees treated with 3 ng imidacloprid (n = 9) during the observation period immediately after treatment. All treated bees returned to the foraging site. (**C**) No verifiable effect was observed for the median time interval spent inside the hive during observation periods. No effect was observed 24 h after treatment.* = p<0.05, ** = p≤0.01, *** = p≤0.001.
(TIF)

Figure S2 **Schematic view of the bee-tunnel cross-section.** (**A**) Cross section of the tunnel designed to ensure passages of the bees with dorsal-surface facing upward. The highest part allows passage of the bee's body, while the side extensions give space to the bee's legs in sideward position. Front view. (**B**) Top view of the two parallel tunnels.
(TIF)

Movie S1 **1^st^ symptom observed after administration of doses ≥3 ng imidacloprid.** Bee showing reduced mobility at the entrance of the feeder compartment.
(AVI)

Movie S2 **2^nd^ symptom observed after administration of doses ≥3 ng imidacloprid.** A near-to-motionless bee sitting on the feeder with occasional trembling and cleaning movements.
(AVI)

Movie S3 **1^st^ symptom observed after administration of doses ≥1 ng clothianidin.** Bee moving around the feeder compartment with an awkwardly arched abdomen.
(AVI)

Movie S4 **2^nd^ symptom observed after administration of doses ≥1 ng clothianidin.** Bee lying on its back with paddling movements unable to return to upright position.
(AVI)
