# Supplementary figures and images for: RFID Tracking of Sublethal Effects of Two Neonicotinoid Insecticides on the Foraging Behavior of Apis mellifera
Source: PLoS One. 2012 Jan 11;7(1):e30023. doi: 10.1371/journal.pone.0030023 (PMC3256199; doi:10.1371/journal.pone.0030023)

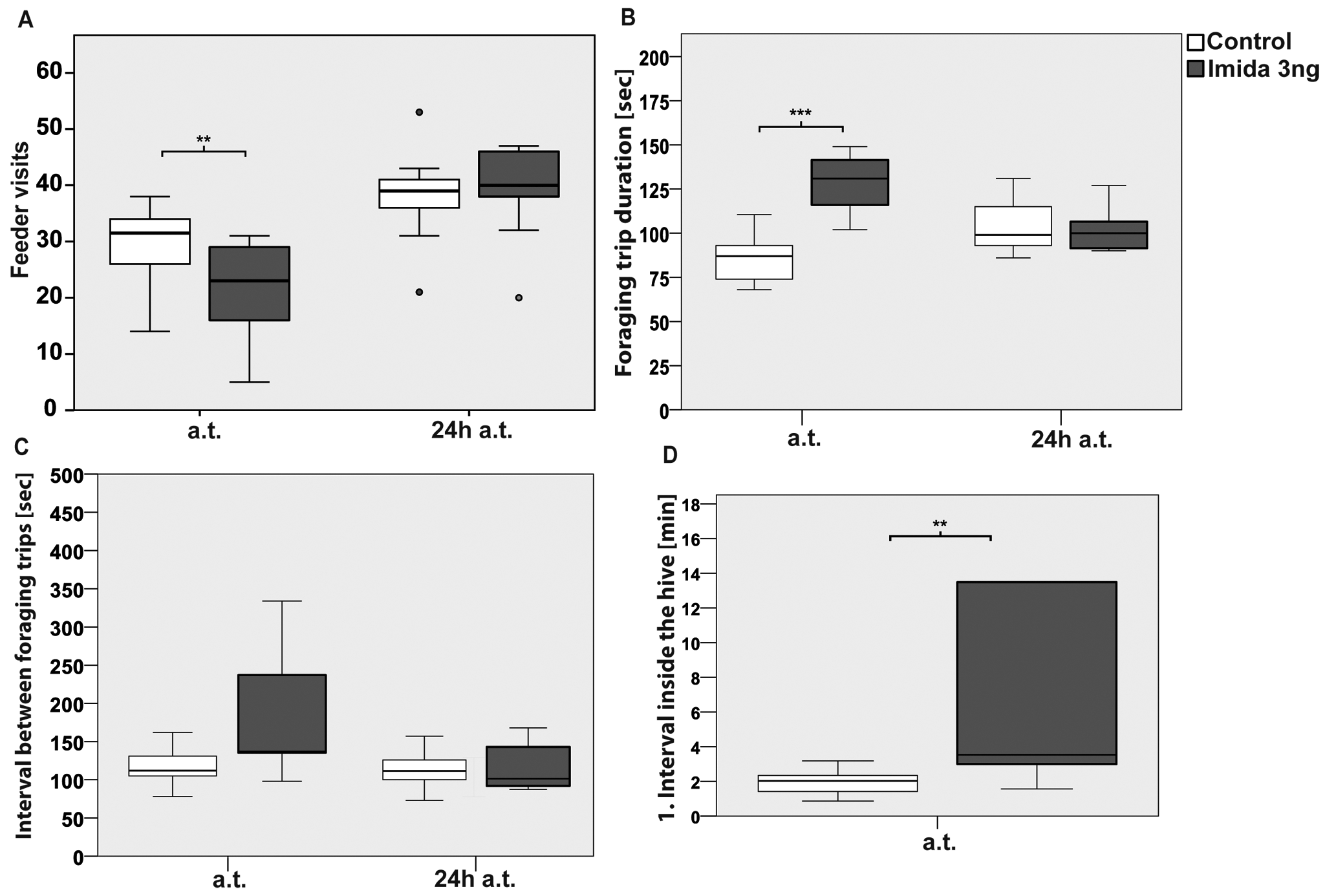

Supplement: Figure S1 — Results of pre-test conducted with a handheld USB-Pen to detect bees at the feeder site. (A) The number of visits at the feeder site was significantly reduced, (B) the median total duration for a single foraging trip and the first time interval spent inside the hive were significantly prolonged compared to the control (n = 10) for bees treated with 3 ng imidacloprid (n = 9) during the observation period immediately after treatment. All treated bees returned to the foraging site. (C) No verifiable effect was observed for the median time interval spent inside the hive during observation periods. No effect was observed 24 h after treatment.* = p<0.05, ** = p≤0.01, *** = p≤0.001. (TIF) [file pone.0030023.s001.tif]

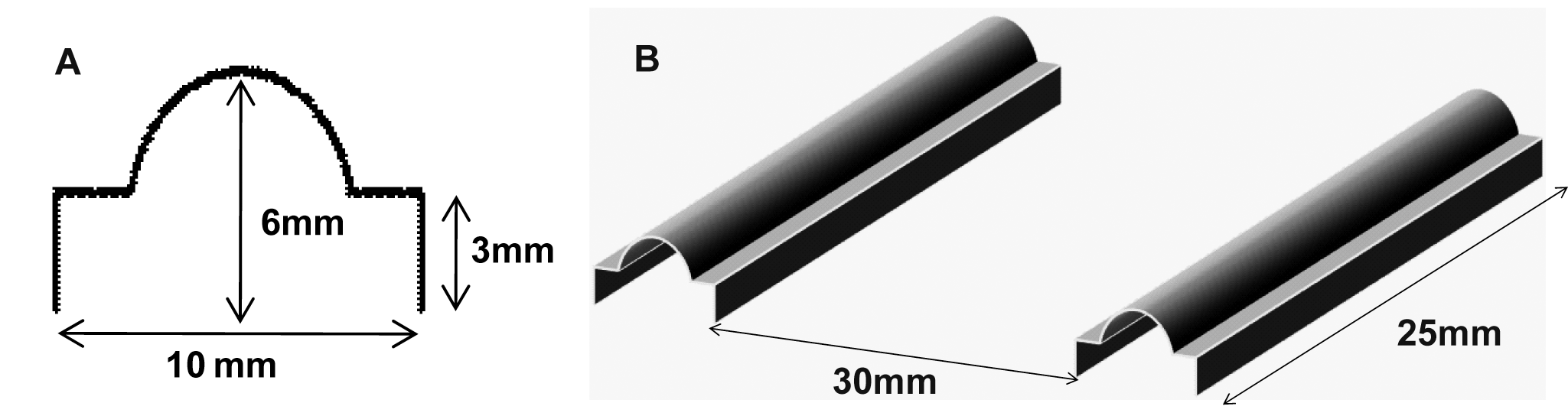

Supplement: Figure S2 — Schematic view of the bee-tunnel cross-section. (A) Cross section of the tunnel designed to ensure passages of the bees with dorsal-surface facing upward. The highest part allows passage of the bee's body, while the side extensions give space to the bee's legs in sideward position. Front view. (B) Top view of the two parallel tunnels. (TIF) [file pone.0030023.s002.tif]
